# Relative effectiveness of a full versus reduced version of the ‘Smoke Free’ mobile application for smoking cessation: an exploratory randomised controlled trial

**DOI:** 10.12688/f1000research.16148.2

**Published:** 2019-01-09

**Authors:** David Crane, Harveen Kaur Ubhi, Jamie Brown, Robert West

**Affiliations:** 1 Department of Behavioural Science and Health, University College London, London, WC1E 6BT, UK; 2 National Centre for Smoking Cessation and Training, Dorchester, DT1 1RD, UK

**Keywords:** smoking cessation, RCT, smartphone application, smoke free

## Abstract

**Background:** Smartphone applications (apps) are popular aids for smoking cessation. Smoke Free is an app that delivers behaviour change techniques used in effective face-to-face behavioural support programmes. The aim of this study was to assess whether the full version of Smoke Free is more effective than the reduced version.

**Methods:**  This was a two-arm exploratory randomised controlled trial. Smokers who downloaded Smoke Free were randomly offered the full or reduced version; 28,112 smokers aged 18+ years who set a quit date were included. The full version provided updates on benefits of abstinence, progress (days smoke free), virtual ‘badges’ and daily ‘missions’ with push notifications aimed at preventing and managing cravings. The reduced version did not include the missions. At baseline the app recorded users’: device type (iPhone or Android), age, sex, daily cigarette consumption, time to first cigarette of the day, and educational level. The primary outcome was self-reported complete abstinence from the quit date in a 3-month follow-up questionnaire delivered via the app. Analyses conducted included logistic regressions of outcome on to app version (full versus reduced) with adjustment for baseline variables using both intention-to-treat/missing-equals smoking (MES) and follow-up-only (FUO) analyses.

**Results:** The 3-month follow-up rate was 8.5% (n=1,213) for the intervention and 6.5% (n=901) for the control. A total of 234 participants reported not smoking in the intervention versus 124 in the control, representing 1.6% versus 0.9% in the MES analysis and 19.3% versus 13.8% in the FUO analysis. Adjusted odds ratios were 1.90, 95%CI=1.53-2.37 (p<0.001) and 1.50, 95%CI=1.18-1.91 (p<0.001) in the MES and FUO analyses respectively.

**Conclusions:** Despite very low follow-up rates using in-app follow up, both intention-to-treat/missing equals smoking and follow-up only analyses showed the full version of the Smoke Free app to result in higher self-reported 3-month continuous smoking abstinence rates than the reduced version.

## Introduction

Smartphone applications (apps) are used by many smokers to aid cessation but currently little evidence exists on their effectiveness. The Smoke Free app ( smokefreeapp.com) is very popular worldwide, with some 4,000 new downloads per day. When it first became available, it was the subject of a trial with users finding and downloading the app from the app store being randomly assigned to a full version or a reduced version. This provided an opportunity to assess whether the full version was more effective than the reduced version in an effectiveness study closely mirroring the real-world scenario of interest. This paper reports the findings from that trial.

The Smoke Free app was developed using behaviour change techniques (BCTs) found in effective behavioural support programmes for smoking cessation ^1^. A description of the app is given in Supplementary File 1. The evidence-based BCTs implemented in the full version of the app were: 1) Supporting identity change: supporting app users to think of themselves as non-smokers, 2) Rewarding abstinence: praise, virtual prizes and showing them how much money they are saving each day of not smoking, 3) Changing routines: advising on ways of avoiding smoking cues by changing routines that involve smoking, and 4) Advising on medication use: promoting the use of one of the evidence-based stop-smoking medicines. These BCTs are designed to increase resolve and prevent, reduce and counter urges to smoke.

Behavioural support delivered via a smartphone could help smokers to stop. Internet-based support has been found in some cases to aid cessation ^2^ and smartphone apps can provide this functionality with the added advantage of being readily accessible at almost any time. Two prospective studies of users of smoking cessation apps ^3, 4^, a randomised controlled trial (RCT) comparing an app with a text messaging intervention ^5^ and two RCTs comparing mindfulness-based apps with other apps ^6, 7^ found self-reported success rates that were higher than would be expected from unaided cessation. One RCT has found that an app acting as a decision aid for smokers interested in stopping smoking resulted in higher 6-month abstinence rates than an information-only app ^8^. Another RCT examined the effectiveness of a set of app components as an aid to cessation in pregnant smokers; engagement with the app was low and no specific components were found to increase short-term self-reported abstinence rates ^9^. To date, no RCTs have been published comparing apps designed to provide ongoing support for quit attempts with unaided quitting, or more intensive versus less intensive versions of an app.

Evaluating the effectiveness of smoking cessation apps versus unaided cessation in RCTs is complicated by the fact that apps are widely available and participants who are randomised to the unaided quitting condition are likely to be motivated to drop out of the study or use one of the many freely available apps. An alternative is to compare full and reduced versions of an app in which the reduced version is sufficiently credible that participants who are randomised to receive it are not motivated to drop out of the study or seek out another app. That was the approach used in the present study.

Another challenge for RCTs of apps is how to address the problem of loss to follow up. With sufficient resources, high follow-up rates can be obtained in such trials ^6, 10^. However, the methods used can lead to problems of generalizability; study engagement processes that involve face-to-face visits, incentives and contracts on the part of participants that may exclude a substantial proportion of the target population. Moreover, the resources required are prohibitive for the kind of agile, iterative evaluation that is required during the development of these interventions, where evaluations need to be undertaken repeatedly ^11^. Automated outcome assessment using the smartphone is low cost and does not require procedures that may undermine generalizability. Despite the fact that it may result in very low follow-up rates that is the approach used in the current study.

In smoking cessation trials it is common practice to use an intention-to-treat approach with participants lost to follow up considered to have resumed smoking ^12^. This may bias effect sizes downwards if loss to follow up occurs for reasons other than relapse to smoking ^13^. Conversely, it may bias effect sizes upwards if the intervention condition leads to higher follow-up rates than the control condition. Only including participants who are successfully followed up may overestimate absolute success rates if participants refuse to engage with follow up because they have resumed smoking, but this would not affect the odds ratio comparing two conditions since this bias would affect both intervention and comparison groups equally. This approach is also immune to bias caused by differential follow-up in intervention and control groups. In practice ‘missing equals smoking’ (MES) and ‘follow-up only’ (FUO) approaches tend to produce very similar odds ratios in smoking cessation RCTs ^14^, though the percentage point difference between conditions varies considerably. Multiple imputation methods are increasingly being used to estimate values for missing data arising from loss to follow up (e.g. Westmaas *et al*. ^15^). However, these are only viable when the proportion of values that are missing is low. To address biases arising from loss to follow up, both the MES and FUO approaches were used in the present study. It may be expected that the true percentage point difference and odds ratios lie somewhere between the estimates provided by these two methods.

Biochemical verification of abstinence is recommended in smoking cessation trials because of psychological pressure to claim abstinence ^12^. However, this is highly resource intensive and may undermine generalizability to smokers who would use an app but not a more intensive interaction. such as this with no personal contact with a counsellor there may be expected to be no greater psychological pressure on participants to falsely claim abstinence in one condition than another’ so reliance on self-reported abstinence should not bias the estimated effect size. Therefore, this study used self-report for outcome assessment.

Duration of follow up is an important consideration in smoking cessation trials. Conventionally, follow-up at least 6 months after the start of an intervention is considered appropriate for definitive trials while shorter durations are acceptable for proof of concept trials ^12^. A recent systematic review of continuous abstinence rates in smoking cessation trials has recently found, however, that rates at 6-month and 12-month follow up can be accurately predicted from findings after 12 weeks ^16^. Loss to follow up may be greater with longer follow up so in the present study participants were followed up 12 weeks after the target quit date.

Thus, this study addressed the question of whether the full version of the Smoke Free app would result in higher 12-week self-reported continuous abstinence rates than a reduced version of the app in smokers downloading the app and using it to set a quit date.

## Methods

### Study design

Participants were individually randomly allocated by the app on a 1:1 ratio to the full or reduced version and followed up automatically by the app 12 weeks after the target quit date to assess the outcome. Randomisation was by a random number generator in the app and generated a 1 or 2 during the registration process independently for each user. This study was not pre-registered because the lead author was not aware of this requirement at the time the data were collected, and so the study must be considered exploratory. We used the CONSORT-SPI checklist in preparing this report ^17^ ( Supplementary File 2). The study was approved by the University of East London Ethics Committee.

### Participants

Participants were not actively recruited and received no financial incentive for taking part. Smokers who downloaded the Smoke Free app between February 2013 and January 2015 were informed by the app that it was being used in an evaluation and asked for permission to use their data for research purposes. The app was available globally but only in the English language. If participants agreed they completed baseline measures and were randomly assigned by a computer-generated random number sequence to be offered a full or reduced version of the app. Consent was given by users by means of the touchscreen on their device. They were then included in the analysis if they met the following criteria: aged 18 years or over, smoked cigarettes at the time of registration (whether daily or non-daily), set only one quit date, and used the app at least once on or after their target quit date. Those users who had started their quit attempt before the date of registration were excluded, and if users registered more than once on the same device (as identified by the device ID) only data from the first registration was used.

Participants were aware that they were taking part in an experiment but were not aware of the details of the condition to which they had not been assigned.

Sample size was determined pragmatically by recruiting from the point where the app was in a form that was stable to the deadline for delivery of the lead author’s project report. A total of 28,112 participants were included in the sample, of whom 14,228 received the full version and 13,884 received the reduced version.

### Intervention and comparator

The full version of the Smoke Free app took smokers through the first month of their quit attempt by helping them maintain their resolve by setting a clear goal, monitor their progress towards that goal and become aware of benefits achieved to date. There were several components: 1) a calculator that tracked the total amount of money not spent on buying cigarettes and the number of cigarettes not smoked; 2) a calendar that tracked the amount of time elapsed since cessation; 3) a scoreboard that awarded virtual ‘badges’ to users for not smoking; 4) progress indicators that informed users of health improvements expected since the start of their quit attempt; and 5) daily missions that were assigned from the start of a user’s quit date for one calendar month.

The daily missions included behaviour change techniques that research has suggested are likely to improve the chances of avoiding and resisting cravings and thereby promote abstinence ^18– 20^. A list of the daily missions can be found in the Supplementary File 1.

The full version of Smoke Free received daily push notifications for one calendar month from the start of their quit date. Users were prompted to open the app to read each day’s mission. The time of the push notification was preset to 8am local time but this could be changed to a time of the user’s preference. For screenshots of the app see Supplementary File 1.

The reduced version of the app was the same as the full version but without the daily missions.

### Measures

After consenting to take part in the experiment, users were asked to provide information on their: age, sex, educational level (high school or secondary school, undergraduate degree, or post-graduate degree), daily cigarette consumption, and time to first cigarette of the day (<5 minutes, 5–30 minutes, 31–60 minutes, >60 minutes) ^21^.

After filling out the baseline questionnaire, users were then requested to record their target quit date which could be any date in the past or future (with those having already quit being excluded from the analysis).

The primary outcome measure was self-reported continuous abstinence up to 12-week follow-up. The app sent users a push notification 12 weeks after the target quit date asking them to open the app and respond to a questionnaire. The app did not send reminder notifications. The questionnaire asked: 1) “Have you smoked at all in the last three months?” to which they could respond: “No, not a puff”, “1–5 cigarettes”, or “More than 5 cigarettes”. Those who responded “not a puff” were considered to be abstinent.

### Analysis

Baseline characteristics of the two groups were compared using chi-squared tests or analyses of variance as appropriate. Outcomes were compared using logistic regression analyses with and without adjusting for all baseline variables. Two analytic approaches were used: 1) MES in which smokers who were lost to follow-up were counted as having smoked, and 2) FUO in which only smokers who responded to the 3-month follow-up were included in the analysis. Odds ratios and 95% confidence intervals were computed, along with p-values.

Data used in the analyses are available as Supplementary File 3 as an SPSS file and the SPSS syntax used to run the analyses is provided in Supplementary File 4. The full data set, including variables not included in the analysis, are provided in Dataset 1.

## Results

**Figure 1.**
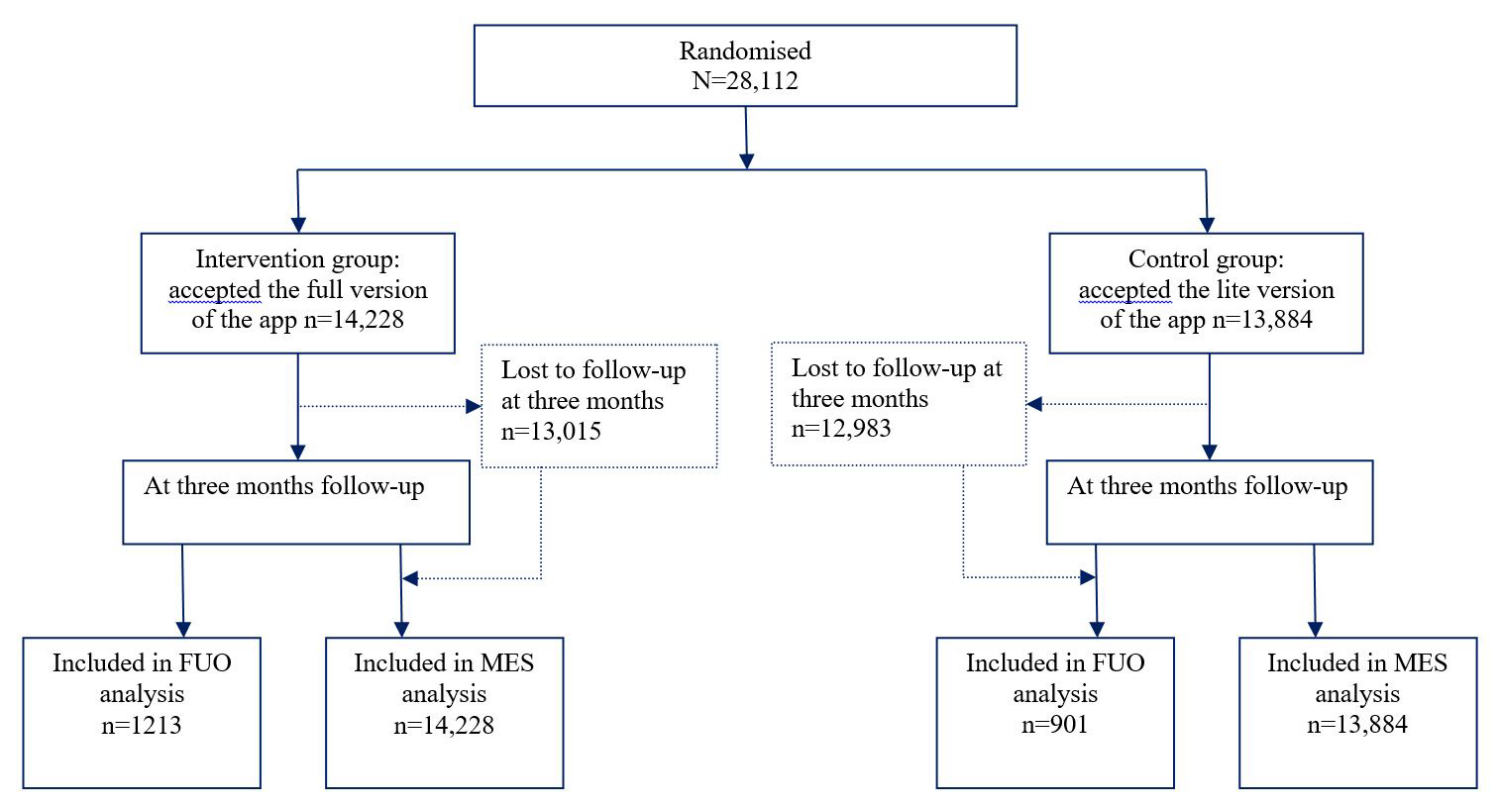
Flow of participants.

Table 1 shows participants’ baseline characteristics and Figure 1 shows the numbers allocated to each group and followed up. Participants who received the reduced version of the app were older, smoked more cigarettes per day, started smoking earlier in the day and were more likely to designate a quit date that was after the date of registration, but the differences were small. Complete data are shown in Dataset 1 ^22^.

**Table 1.** Baseline characteristics of the study sample.

Variable	Reduced version	Full version	Total
Device type:			
Android, N (%)	1,075 (7.7)	1,044 (7.3)	2,119 (7.5)
iOS, N (%)	12,809 (92.3)	13,184 (92.7)	25,993 (92.5)
Age, mean years (SD) *	29.1 (9.4)	28.7 (9.0)	28.9 (9.2)
Sex			
Female, N (%)	6,769 (48.8)	7,015 (49.3)	13,784 (49.0)
Male, N (%)	7,115 (51.2)	7,213 (50.7)	14,328 (51.0)
Educational level			
School only, N (%)	8,734 (62.9)	8,949 (62.9)	17,683 (62.9)
Undergraduate, N (%)	3,477 (25.0)	3,598 (25.3)	7,075 (25.2)
Postgraduate, N (%)	1,673 (12.0)	1,681 (11.8)	3,354 (11.9)
Quit date			
Day of registration, N (%)	9,514 (68.5)	9,915 (69.7)	19,429 (69.1)
After registration, N (%)	4,370 (31.5)	4,313 (30.3)	8,683 (30.9)
Cigarettes per day, mean (SD) *	14.8 (7.6)	14.6 (7.4)	14.7 (7.5)
Time to first cigarette *			
<6 minutes, N (%)	3,679 (26.5)	3,569 (25.1)	7,248 (25.8)
6–30 minutes, N (%)	4,374 (31.5)	4,350 (31.8)	8,904 (31.7)
31–60 minutes, N (%)	2,788 (20.1)	2,896 (20.4)	5,684 (20.2)
>60 minutes, N (%)	3,043 (21.9)	3,233 (22.7)	6,267 (22.3)

*p<0.05 for comparison between groups, not adjusted for number of comparisons.

Of the participants, 2,114 (7.5%) were followed up (full version 1,213, 8.5%, reduced version 901, 6.5%). In the MES analysis 1.6% (n=234) of the participants in the intervention group and 0.9% (n=124) of the participants in the control group reported as being abstinent from smoking (unadjusted Odds ratio=1.86; 95% CI=1.49-2.31; p<0.001; risk difference 0.7%). In the FUO analysis, 19.3% in the intervention group and 13.8% in the comparison group reported being abstinent (unadjusted Odds Ratio=1.50; 95% CI=1.18-1.90; p<0.001; risk difference 5.5%).

Table 2 shows the results from logistic regression analyses with app version and all baseline variables entered together. In both the MES and FUO analyses, participants randomized to the full version of the app had higher odds of reporting successful abstinence with odds ratios almost identical to the unadjusted regression analyses. A number of baseline variables also predicted reported abstinence. Older participants were more likely to report abstinence, while those using Android (versus iOS devices) and those whose quit date was after (versus on) the date of registration were less likely to report abstinence. In the MES analysis, participants whose first cigarette of the day was more than 5 minutes from waking were more likely to remain abstinence that those who smoked within 5 minutes of waking, but the difference was not statistically significant for those smoking their first cigarette more than 60 minutes from waking.

**Table 2.** Results of adjusted logistic regression analyses of outcome on to treatment group and baseline variables.

Predictor variable	Missing equals smoking analysis	Follow-up only analysis
Treatment group		
Reduced version	Reference	Reference
Full version	1.90 (1.52-2.37) *	1.50 (1.18-1.91) *
Device type		
iOS	Reference	Reference
Android	0.25 (0.12-0.50) *	0.29 (0.14-0.60) *
Age, years	1.05 (1.04-1.06) *	1.03 (1.02-1.04) *
Sex		
Male	Reference	Reference
Female	1.23 (0.99-1.52)	1.00 (0.79-1.27)
Educational level		
School only	Reference	
Undergraduate	1.06 (0.83-1.35)	0.93 (0.71-1.22)
Postgraduate	1.01 (0.73-1.39)	1.10 (0.77-1.58)
Quit date		
Day of registration	Reference	Reference
After registration	0.43 (0.33-0.57) *	0.69 (0.51-0.93) *
Cigarettes per day	1.01 (1.00-1.03)	1.00 (0.99-1.02)
Time to first cigarette		
<6 minutes	Reference	Reference
6–30 minutes	1.61 (1.21-2.14) *	1.36 (0.99-1.86)
31–60 minutes	1.53 (1.10-2.15) *	1.19 (0.82-1.73)
>60 minutes	1.27 (0.88-1.83)	0.99 (0.66-1.48)

*p<0.05 for linear trend or comparison with reference.

Full de-identified data from each study participant, including download dates, quitting dates and all other data input into the app Click here for additional data file. Copyright: © 2019 Crane D et al. 2019 Data associated with the article are available under the terms of the Creative Commons Zero "No rights reserved" data waiver (CC0 1.0 Public domain dedication).

## Discussion

In both the MES and FUO analyses the full version of the Smoke Free app produced higher self-reported abstinence rates than the reduced version 12 weeks after the target quit date. The odds ratios were 1.86 and 1.50 in the MES and FUO analyses respectively, and the percentage point differences between full and reduced versions were 5.5% and 0.7%. 

Even with very low follow-up rates the study found a small but clear advantage to the full version of the app which may be attributed to the inclusion of the daily missions. The effect size in terms of odds ratios was similar to, or slightly lower, than was found in the only published RCT to date to have found a clear effect of a smoking cessation app. This effect is on top of whatever effect the reduced version of the app may have had. Even in the follow-up only analysis the abstinence rates were relatively low, and lower than is found in studies involving face to face support or pharmacotherapy ^23^. Therefore, this app should not be regarded as a substitute for those forms of support. It is possible that this app could increase abstinence rates in smokers using such forms of support but this remains to be tested.

The fact that an intervention effect was found in the FUO analyses indicates that it was not due to bias arising from differential loss to follow up. The fact that adjusting for baseline variables that are predictive of successful cessation did not influence the odds ratios adds confidence that the results were not due to smokers who found it easier to stop being more likely to be followed up in the intervention condition.

The lower success rate in participants using Android versus iOS devices needs to be investigated further. It was not explained by other baseline characteristics measured in this study. It may reflect the fact that Android users tend to have lower socioeconomic position or it could be that some of the devices do not have as high usability, e.g. in terms of screen size or resolution.

Strengths of the current study are the large sample size, the fact that it assessed an app that is very popular and therefore needs to be evaluated, and high generalizability to the population of interest, i.e. smokers finding the app on apps stores. Limitations are the very low follow-up rate, use of self-reported abstinence, the relatively short follow-up duration and the absence of process measures to assess what mediated the intervention effect. The generalisability of the study is limited by the low follow-up rate and to smokers who are willing to download an app such as this for help with stopping smoking.

Research continues into apps to support smoking cessation ^24– 29^, with improvements in technology providing new opportunities for intervention content, such as virtual reality and use of wearable devices. The popularity of the Smoke Free app should make it a useful vehicle for testing innovations in smoking cessation support, building on the version of the app tested in this study. Research is needed into improving follow-up rates without compromising generalizability and within the resource constraints operating on companies and research groups seeking to build incrementally on app performance.

In conclusion, the full version of the Smoke Free smartphone app appears to have small effect in improving 12-week abstinence rates in smokers trying to quit. This provides a basis for building a programme of incremental improvement in effectiveness.

## Data availability

The data referenced by this article are under copyright with the following copyright statement: Copyright: © 2019 Crane D et al.

Data associated with the article are available under the terms of the Creative Commons Zero "No rights reserved" data waiver (CC0 1.0 Public domain dedication). 

**Dataset 1. Full de-identified data from each study participant, including download dates, quitting dates and all other data input into the app.** Data are available in SAV and CSV formats. DOI: https://doi.org/10.5256/f1000research.16148.d218541 ^22^.

## Supplementary material

**Supplementary File 1. Details on the Smoke Free app, including each day’s mission.**

**Supplementary File 2. Completed CONSORT-SPI checklist.**

**Supplementary File 3. Data used in the analyses.**

**Supplementary File 4. SPSS command syntax for the analyses.**
