# Supplementary material for: Relative effectiveness of a full versus reduced version of the ‘Smoke Free’ mobile application for smoking cessation: an exploratory randomised controlled trial
Source: F1000Res. 2019 Jan 9;7:1524. Originally published 2018 Sep 21. [Version 2] doi: 10.12688/f1000research.16148.2 (PMC6347038; doi:10.12688/f1000research.16148.2)

# Supplementary file S1

## Smoke Free Missions

### Day 1

These missions are designed to help you resist cravings, fall out of love with smoking and feel proud about being a non-smoker. There are 31 in total and some may seem a little strange. Feel free to substitute any ones you don’t like for ones you do, but make sure to do something each day. It’s the process that works.

One of the golden rules of stopping smoking is never to allow yourself even another puff of tobacco smoke. There may be times in the future when you'll think, oh just one won’t hurt. The problem is, it's never just one. The first invariably leads to a second and soon you're back smoking again.

Today’s mission is to make your mantra “Not one more puff, no matter what”. Repeat over and over again, not just today but whenever you can in the future. Make a promise to yourself that you will never touch tobacco again.

### Day 2

Today’s mission is designed to provide a tangible sense of progress and to give you a reward for the work you're doing.

How much were you spending on smoking each day? Find a jar and put in that amount for yesterday and today. Repeat tomorrow and for the remainder of these missions. Jars are good because you can see the money increasing. Doing it to the penny is good because it demonstrates commitment.

It’s best if you keep the jar somewhere you see it.

### Day 3

A craving is not a command and neither cravings nor moods last forever. Your mission today is to let them pass. Don’t try and change them, don’t judge them and don’t judge yourself for having them. Just don’t respond to them, they’ll pass.

Remember, cigarette cravings are very different to the hunger for food. If we don’t eat we will become more hungry. Whereas, if we don’t smoke our cravings will become less severe.

If you find that lettings cravings pass doesn’t work do a search online for other techniques that people have found effective. Millions of people have successfully resisted cravings, what worked for them could work for you too.

### Day 4

Today is about future preparation and the well-tested idea that the best way to deal with a difficult situation is to have an advance plan for it. What are the triggers that might temp you to smoke again? Big ones are often the regular times you smoke (on waking or after a meal for example) when drinking, being in stressful situations, or when feeling anxious.

Your mission is to imagine three of these situations, for example: having an argument with your partner; being in the pub on Friday night; getting an unexpected bill. Then to write down an if-plan for dealing with each one. For example, you might say that “IF I’m tempted to smoke after drinking alcohol THEN I’ll say to myself: Not another puff, no matter what.

Search for implementation intentions, see pre-quit mission -3 or see the help tab of this app for more info on this approach. It can be highly effective.

Keep putting money in your jar.

### Day 5

It can be good to spend time in the company of quitters and if that’s hard to do in person the internet can help. A quick Google will reveal many different smoking cessation sites but one that’s particularly good is [reddit.com/r/stopsmoking](http://reddit.com/r/stopsmoking).

Your mission today is to visit it (or a site of your choosing) and read 10 posts from people in the early stages of quitting. Evidence suggests that other people can really help us stay smoke free, even if that just means realising you’re not the only one going through this.

Don’t forget to put money in your jar.

### Day 6

What are the five biggest benefits you’ve found from giving up smoking? Write them down below and come back to this mission if ever you’re tempted to smoke again.

### Day 7

It’s almost a week since your quit date – well done for getting this far! We can often get through the first few days on willpower alone. But willpower is a depletable resource and when you’re running low, resisting the temptation to smoke can be difficult.

The good news is that you can increase willpower with treats. That might mean eating something sweet, giving yourself permission not to do a piece of work, getting more sleep, or many other things besides.

Your mission today is to search online for increasing willpower – both to better understand how it can be depleted and also what you can do to increase it. Don’t worry that treating yourself now will lead to more problems in the future. Your current goal is to stop smoking. Do that and your feelings of self-control will increase, and you can use increased belief in your inner strength to cut down on the doughnuts later on.

### Day 8

It’s time to feel proud about what you’ve achieved. Every time you think about smoking pause, and breathe deeply three times in a row. See if you can let a little smile come across your face. Remember, you are now a non-smoker.

### Day 9

It’s time to try the Not one more puff mantra again (it was last found in Mission 1). It might feel silly to repeat a mantra over and over again but evidence suggests that having a golden rule like this in place makes you less likely to relapse later.

So your mission today is to repeat to yourself “Not one more puff, no matter what” ten times at ten different points during the day. Bonus points if you can do it twenty times.

### Day 10

Tell five friends you've quit smoking and feel proud as you do. Doing it online is okay.

Feel free to use any of the share options you’ll find on various screens of the app.

Are you still putting money in your jar?

### Day 11

Why are you glad you’re a non-smoker? Recall all the reasons you wanted to quit smoking and add any new ones you’ve thought of since. Spend a minute reflecting on each of them and as you do, breathe deeply and let your chest swell with pride. You’ve kept this promise to yourself for ten days now. Did you think you'd make it this far?

### Day 12

Hopefully your cravings will have lessened significantly by now. However, they can flare up at any time and you will need a way of resisting them if you’re to carry on being successful with your quit.

So what will you tell yourself if you have a strong desire to smoke? How will you deal with it? How will you stay strong? Go back to the implementation intentions mission from Day 4, remember what you wrote then and try to write down ideas for three more situations now.

### Day 13

Spend five minutes reading posts on [reddit.com/r/stopsmoking](http://reddit.com/r/stopsmoking) (or your favourite stop smoking site). Use these sites to gain more knowledge about the process of quitting and to understand that other people have been and are going through what you're going through.

Repeat five times at various points in the day, or any time you have a craving. For bonus points, reply to a post or two, or post something of your own.

### Day 14

Imagine totally crushing a packet of cigarettes. Stomp on it, tear it to bits, destroy the thing. And feel angry as you do. Then throw the rubbish as far away as you can. Bonus points if you do it five times at various points today.

### Day 15

Breathe in deeply three times, smile and think to yourself, I’ve done two weeks smoke free. Bonus points if you repeat this five times today. Double bonus points if you repeat it ten times.

### Day 16

What are the benefits you’ve gained from giving up smoking? What feels different, what can you do differently now?

Spend a couple of minutes comparing your life before quitting to how it is now. Try to come up with at least three things you think are better and write them down below. The more benefits you can come up with the less likely you’ll return to that disgusting and deeply harmful habit.

### Day 17

Is there anyone who doesn't know you've given up smoking? Make today the day you tell them. Chances are they'll feel happy for you and proud of your achievements. At the very least, telling others you've quit makes it less likely you'll relapse.

### Day 18

What do you think when you see a smoker now? Are you envious or delighted you’re not one of them? How does the smell of a smoke seem on clothes, in rooms or cars? How do you feel about being different to a smoker?

Changing our smoking identity is an important step in moving away from cigarettes. If you’re glad, or can become glad, you’re an ex-smoker the temptation to return to what is actually a pretty disgusting habit will be less.

### Day 19

Write below a list of all the things that have worked so far and add a little detail about why you think they did. Bonus points if you post them to [reddit.com/r/stopsmoking](http://reddit.com/r/stopsmoking) (or your favourite stop smoking site).

### Day 20

Find five minutes in your day. Get into a comfy chair, lean back and put your feet up. Think about how well you’ve done. Do that chest swelling thing again. Bonus points if you time yourself.

### Day 21

What is the furthest you've gone to have a smoke? Have you fished out butts from the bin, stood smoking in the pouring rain, driven miles in the middle of the night to get a new pack?

Spend a couple of minutes reflecting on the way cigarettes used to have a hold over you and all the things you used to do to feed your addiction. As you do, take pride in the fact that you don't need to do that any longer.

### Day 22

Well done for making it three weeks smoke free. Today’s mission is to make a contract with yourself never to smoke again. This needs to be written down and for best results it should be witnessed by someone else too. If you use Facebook post something like: I hereby commit to myself and to you that I will never smoke even another puff of a cigarette again, no matter what. Or write it on paper and sign it in front of your partner or a friend.

### Day 23

Go to your favourite stop smoking site and help three people starting or struggling with their quit. Share what worked for you, what didn’t, how you feel at this stage, what the benefits have been, or anything else that comes to mind. There are plenty of people in the early stages of quitting who will be very appreciative of your knowledge.

### Day 24

You’ve done brilliantly to get this far but remember, the craving to smoke can flare up at any time and our brain has all kinds of ways of convincing ourselves that just one cigarette won't hurt.

Do you recall the Not a puff mantra from mission 1? Let’s change that a little. Instead of saying “Not a puff, no matter what” try saying: “I don’t smoke” See if you can repeat this to yourself 100 times today. And do so confidently, and with power.

Feel free to keep saying “Not a puff, no matter what” if you prefer.

### Day 25

How have you coped with the craving to smoke? What have you done or told yourself that has worked? What techniques have you tried? Spend a few minutes writing down and reflecting on the strategies you’ve adopted so you can be clear about the ones you know are best for you.

### Day 26

Go to the dashboard and take a look at how many cravings you’ve resisted so far. Did you ever imagine you could resist that many? Let the knowledge of how well you’ve done sink in for a minute. Chances are you’ll have faced down more cravings in the past 25 days than you’ll face in the next six months.

Haven’t used the diary to record cravings? No problem. Bring to mind the day you quit and the days following. Remember how hard it was to resist cravings then and ask yourself if it’s easier now. Hopefully the answer will be yes.

If you’re still struggling, firstly congratulate yourself on staying strong for so long. Next, go to a stop smoking site and find other people who still experience strong cravings. They will have ideas of how you can cope.

### Day 27

If you wear a watch put it on the opposite wrist to normal. If you don’t wear a watch put on a fresh piece of jewellery; a ring, a chain, a bracelet, something you haven’t worn for a while. Each time you notice it say: “I don’t smoke”. Feel proud as you do.

If you’re already out of the house try writing a note on the back of your hand, or colouring part of your finger. These little tricks will help you remember to repeat the mantra.

### Day 28

The first month is usually the hardest but you still need to remain vigilant to ensure you deal with any cravings that arise. Do you remember the implementation intentions from Mission 4 and 12? Bring them to mind now and make sure you know what you’ll do when your guard is down and the urge to smoke appears again.

If you don't have a solid plan for dealing with the triggers, now would be a great time to write one.

### Day 29

Who's the person that's most proud about your non-smoking? Send them a message or give them a call to thank them for how their support has helped. If you've not told anyone yet, or if no-one is particularly supportive, try posting to a stop-smoking site. There will be loads of supportive people there.

### Day 30

Find five minutes in your day. Get into a comfy chair, lean back and put your feet up. Think about how well you’ve done. Do that chest swelling thing again. Bonus points if you time yourself.

Bonus points if you do it twice today

### Day 31

Take all the money you’ve put into your smoke free jar and spend it on a present for yourself. Giving up smoking is a great achievement and you should feel very proud of what you’ve done. Make that feeling stick by buying yourself a nice rewarding gift.

You’ve done brilliantly to get this far but remember, you still have to resist each and every craving if you’re to stay smoke free. We’re still here to help and you’ll find a few missions to get you through the next month. But today is all about celebrating your achievement. Well done you.

# Screenshots

### App dashboard

**All participants**

Calculates money saved and cigarettes not smoked

Calendar tracks amount of time smoke free


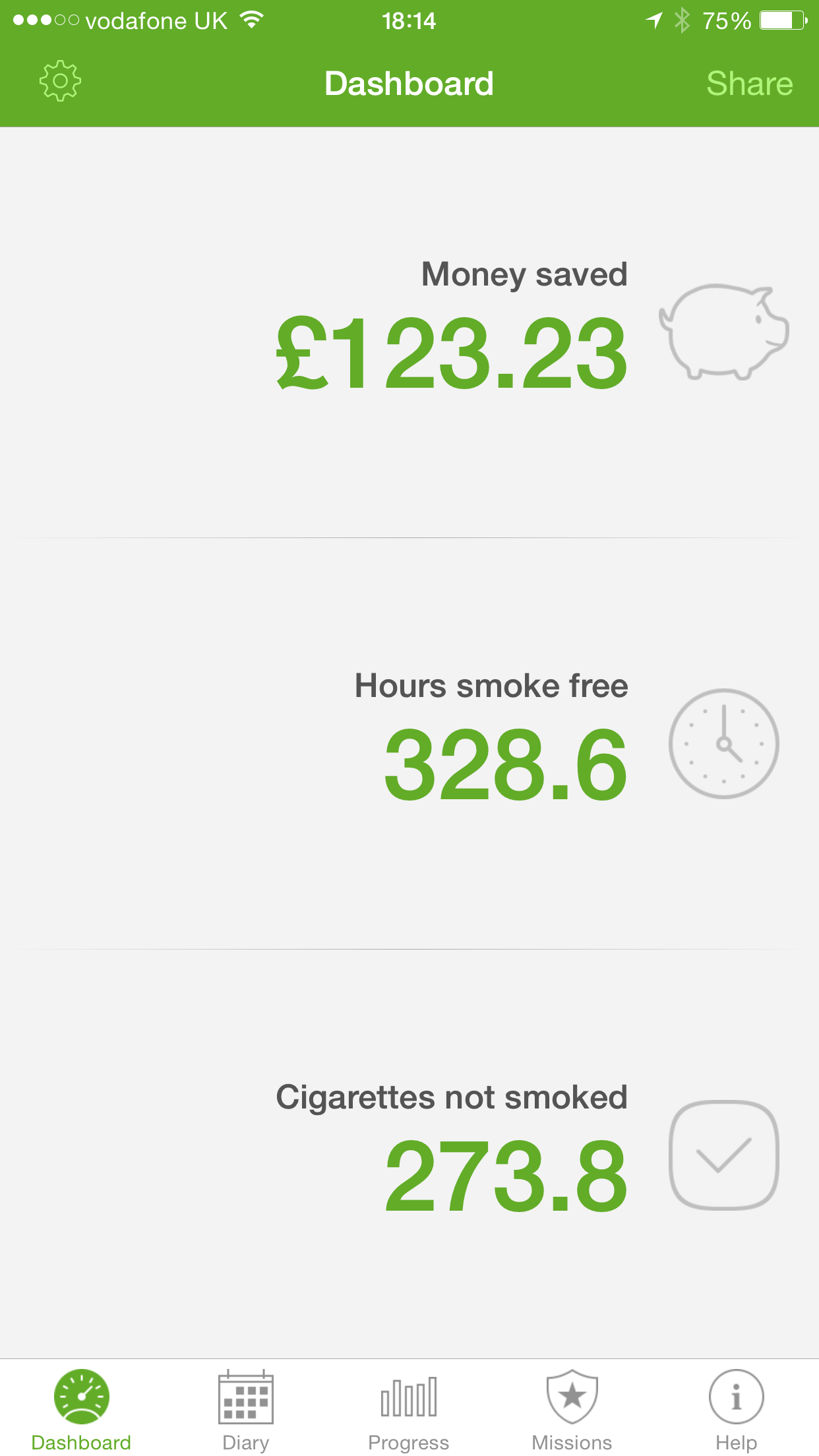


### Virtual Badges

**All participants**

Rewards users for their non-smoking achievements


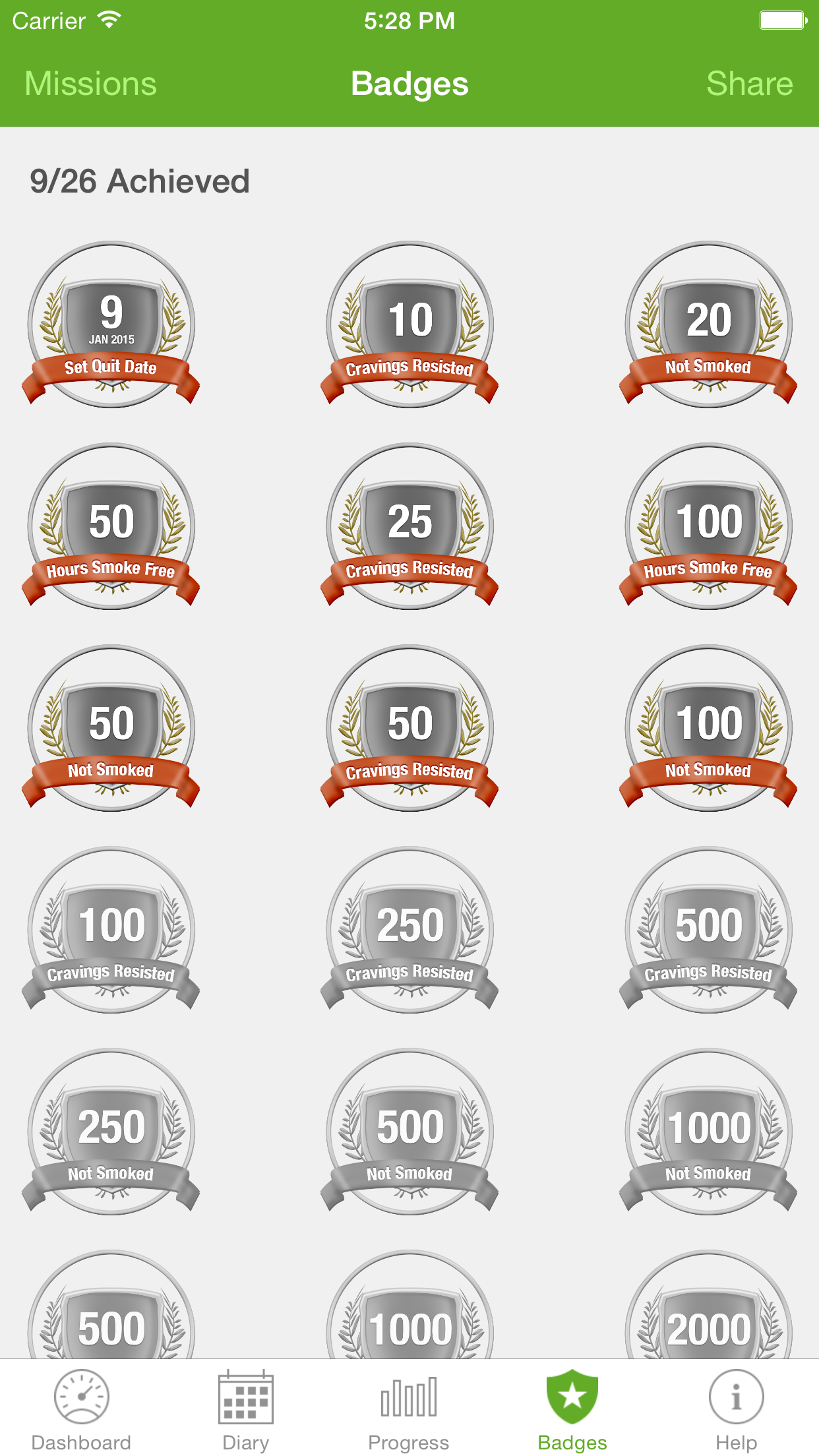


### Health progress indicators

**All participants**

Shows health improvements made since the start of the quit attempt


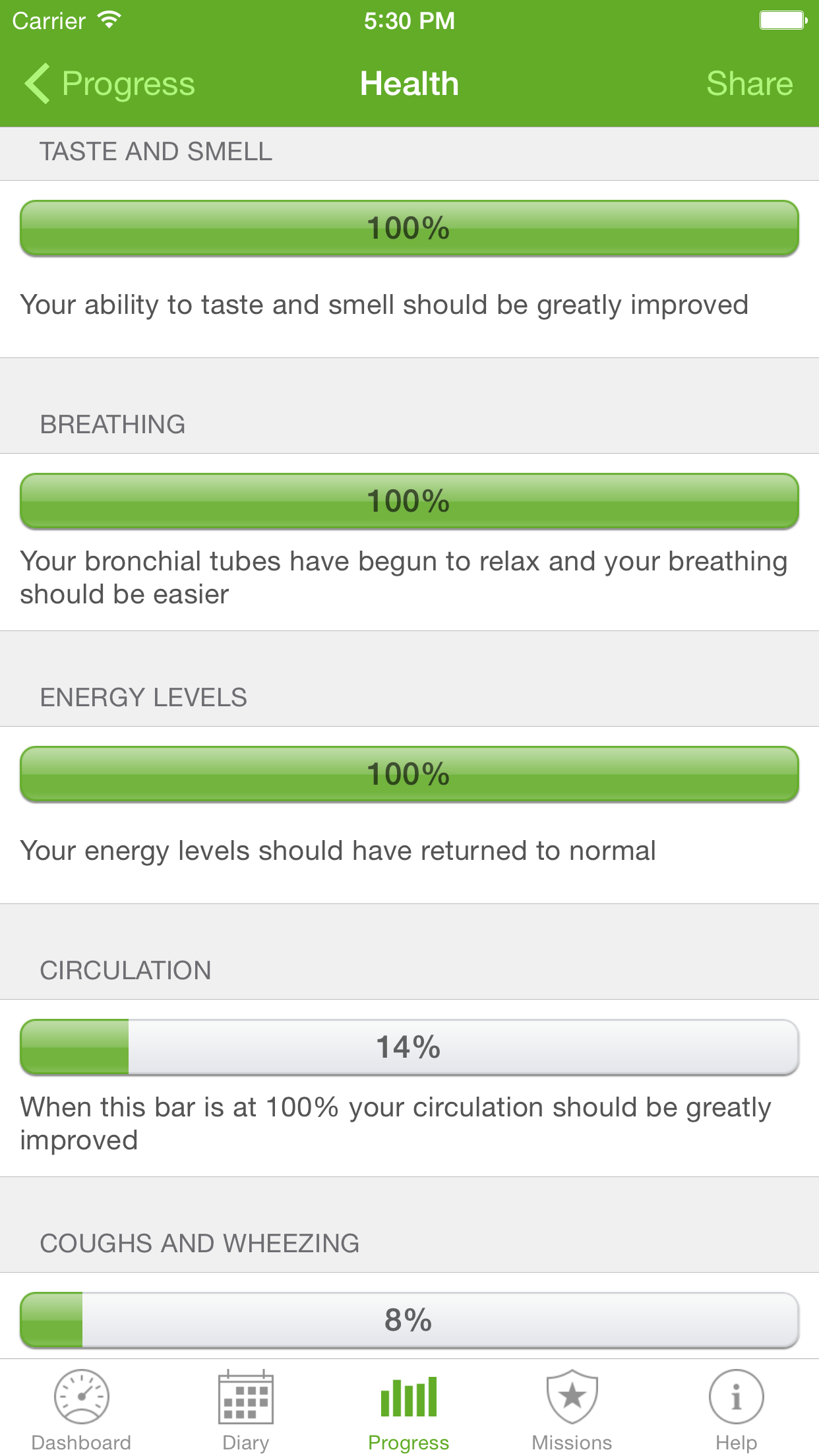


### Missions

**Experimental version only**

Example mission provided to participants given the experimental version of the app.


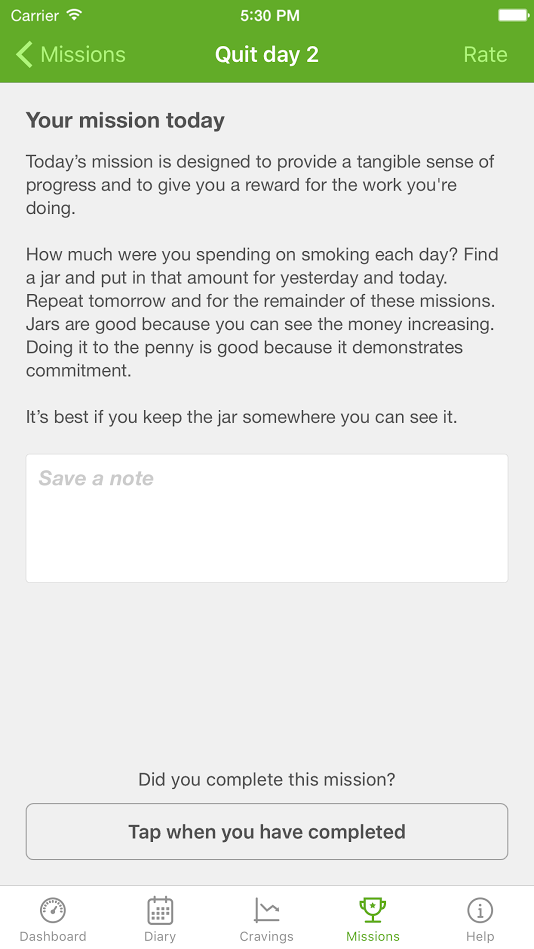

Supplement: Supplementary file 2 [file f1000research-7-19461-s0001.tgz › 891e1ed5-c4b7-427f-ae00-e7c6b66012bd_Supplementary_File_1_App_details.docx]
