# Supplementary material for: Relative effectiveness of a full versus reduced version of the ‘Smoke Free’ mobile application for smoking cessation: an exploratory randomised controlled trial
Source: F1000Res. 2019 Jan 9;7:1524. Originally published 2018 Sep 21. [Version 2] doi: 10.12688/f1000research.16148.2 (PMC6347038; doi:10.12688/f1000research.16148.2)
Supplement: Supplementary file 3 [file f1000research-7-19461-s0002.tgz › c934ef9f-5316-40b3-91a1-afe20f0f903a_Supplementary_File_2_CONSORT_SPI_Checklist.docx]

CONSORT SPI Checklist

| **Title and abstract** |  |
| --- | --- |
| Identification as a randomised trial in the title**^§^** | x |
| Structured summary of trial design, methods, results, and conclusions (for specific guidance, see CONSORT for Abstracts)**^§^** | x |
| **Introduction** |  |
| Scientific background and explanation of rationale**^§^** | x |
| Specific objectives or hypotheses**^§^** | x |
| If pre-specified, how the intervention was hypothesised to work | x |
| **Methods** |  |
| Description of trial design (such as parallel, factorial) including allocation ratio**^§^** | x |
| Important changes to methods after trial commencement (such as eligibility criteria), with reasons | n/a |
| Eligibility criteria for participants**^§^** | x |
| When applicable, eligibility criteria for settings and those delivering the interventions | n/a |
| Settings and locations where the data were collected | x |
| The interventions for each group with sufficient details to allow replication, including how and when they were actually administered | x |
| Extent to which interventions were actually delivered by providers and taken up by participants as planned | n/a |
| Where other informational materials about delivering the intervention can be accessed | x |
| When applicable, how intervention providers were assigned to each group | n/a |
| Completely defined pre-specified outcomes, including how and when they were assessed | x |
| Any changes to trial outcomes after the trial commenced, with reasons | n/a |
| How sample size was determined**^§^** | x |
| When applicable, explanation of any interim analyses and stopping guidelines | n/a |
| Method used to generate the random allocation sequence | x |
| Type of randomisation and details of any restriction (such as blocking and block size)**^§^** | x |
| Mechanism used to implement the random allocation sequence, describing any steps taken to conceal the sequence until interventions were assigned**^§^** | x |
| Who generated the random allocation sequence, who enrolled participants, and who assigned participants to interventions**^§^** | x |
| Who was aware of intervention assignment after allocation (for example, participants, providers, those assessing outcomes), and how any masking was done | x |
| Statistical methods used to compare group outcomes | x |
| How missing data were handled, with details of any imputation method | x |
| Methods for additional analyses, such as subgroup analyses, adjusted analyses, and process evaluations | n/a |
| **Results** |  |
| For each group, the numbers randomly assigned, receiving the intended intervention, and analysed for the outcomes | x |
| Where possible, the number approached, screened, and eligible prior to random assignment, with reasons for non-enrolment | n/a |
| For each group, losses and exclusions after randomisation, together with reasons | x |
| Dates defining the periods of recruitment and follow-up | x |
| Why the trial ended or was stopped | x |
| A table showing baseline characteristics for each group | x |
| Include socioeconomic variables where applicable | x |
| For each group, number included in each analysis and whether the analysis was by original assigned groups**^§^** | x |
| For each outcome, results for each group, and the estimated effect size and its precision (such as 95% confidence interval)**^§^** | x |
| Indicate availability of trial data | x |
| For binary outcomes, presentation of both absolute and relative effect sizes is recommended | x |
| Results of any other analyses performed, including subgroup analyses, adjusted analyses, and process evaluations, distinguishing pre-specified from exploratory | x |
| All important harms or unintended effects in each group (for specific guidance, see CONSORT for Harms) | n/a |
| **Discussion** |  |
| Trial limitations, addressing sources of potential bias, imprecision, and, if relevant, multiplicity of analyses | x |
| Generalisability (external validity, applicability) of the trial findings | x |
| Interpretation consistent with results, balancing benefits and harms, and considering other relevant evidence | x |
| **Important information** |  |
| Registration number and name of trial registry | n/a |
| Where the full trial protocol can be accessed, if available | n/a |
| Sources of funding and other support, role of funders | x |
| Declaration of any other potential interests | x |
| **Stakeholder involvement** |  |
| Any involvement of the intervention developer in the design, conduct, analysis, or reporting of the trial | x |
| Other stakeholder involvement in trial design, conduct, or analyses | n/a |
| Incentives offered as part of the trial | x |
