# Supplementary material for: Relative effectiveness of a full versus reduced version of the ‘Smoke Free’ mobile application for smoking cessation: an exploratory randomised controlled trial
Source: F1000Res. 2019 Jan 9;7:1524. Originally published 2018 Sep 21. [Version 2] doi: 10.12688/f1000research.16148.2 (PMC6347038; doi:10.12688/f1000research.16148.2)
Supplement: Peer review history

# Peer review: Referee response for version 2

**DOI:** 10.5256/f1000research.19461.r42820

**Published:** 2019-01-24

**Authors:** Erica Cruvinel

**Affiliations:** 1 Preventive Medicine and Public Health Department, University of Kansas Medical Center, Kansas City, Kansas, USA

The authors addressed all comments. I don't have any other suggestions.

I have read this submission. I believe that I have an appropriate level of expertise to confirm that it is of an acceptable scientific standard.

# Peer review: Referee response for version 2

**DOI:** 10.5256/f1000research.19461.r42819

**Published:** 2019-01-22

**Authors:** Jonathan Bricker

**Affiliations:** 1 Fred Hutchinson Cancer Research Center, Seattle, WA, USA

I accept their revision and have no further comments.

I have read this submission. I believe that I have an appropriate level of expertise to confirm that it is of an acceptable scientific standard.

# Peer review: Referee response for version 1

**DOI:** 10.5256/f1000research.17634.r39534

**Published:** 2018-10-23

**Authors:** Jonathan Bricker

**Affiliations:** 1 Fred Hutchinson Cancer Research Center, Seattle, WA, USA

This manuscript reports on the results of a fully automated RCT that compared the Smoke Free app with and without 30 days of behavior change technique skills for quitting and preventing relapse ("missions").

 Major strengths:

1. The scientific premise of the study is strong. Determining the added value of behavior change techniques for smoking cessation is useful in any context (not just apps) so the study affords a real-world opportunity to test the techniques' potential effectiveness.
2. Testing the effectiveness of a widely utilized app in real-world conditions of consumers actually downloading to help them quit smoking is valuable from the point of view of implementation research. And perhaps that is the best context from which to frame this study--a real world implementation study.
3. Novelty. There is a dearth of studies comparing apps for smoking cessation so the study adds to a small and important literature.

Major weaknesses:

1. Trial was not registered. This is a very serious weaknesses that impacts ethical obligations to participants, research community, and publication bias. Reputable journals would not even consider reviewing this paper knowing that is was not registered. The authors are aware of this weakness and I have no reason to believe they acted intentionally unethically. Nonetheless, it would be appropriate not to title the study a randomized trial or use that term in the abstract. Instead, I recommend the authors use the same term employed in methods section of the study design: "Exploratory Study." The authors themselves call it that. Now they just need to make it clear up front.
2. Very low retention rate (7.5%) that appears to imbalanced by a relative rate of 24% between arms. This is a very unfortunate weakness that could have been avoided with some small and immediate incentives given that only one question is asked in the follow-up survey. Nonetheless, it is what it is. The level of bias from this extent of missing data (92.5%) cannot be overcome with imputation methods. And the argument that the relative difference (not absolute quit rate) is what matters for this study is undermined by the imbalanced retention rates. With 24% more data in one arm than the other, it very possible that the difference in quit rates is simply driven by the difference in retention rates.

Moderate weaknesses:

1. The main outcome of continuous abstinence after baseline is biased by differences in quit dates and simply does not give people enough time to even reach their quit date before they would already be counted a smoker. Thus, the actual quit rates could be higher. Its hard to say with this outcome. 
2. Generalizablity is overstated for two reasons. The low retention rate makes the sample highly biased toward the most motivated people who are most likely to be reporting that they quit. Comparing the baseline characteristics of all those enrolled vs only those completed the outcome survey item would be very important and instructive about bias. The second reason it is overstated is that the sample is limited to those who have already chosen an app and this particular app to help them quit smoking. The sample age (29) is young for a mhealth smoking study, which typically is about age 40. 

I have read this submission. I believe that I have an appropriate level of expertise to confirm that it is of an acceptable scientific standard, however I have significant reservations, as outlined above.

# Response

**Published:** 2019-01-08

**Authors:** Robert West

**Affiliations:** University College London, UK

Major weaknesses

1. Trial was not registered. This is a very serious weaknesses that impacts ethical obligations to participants, research community, and publication bias. Reputable journals would not even consider reviewing this paper knowing that is was not registered. The authors are aware of this weakness and I have no reason to believe they acted intentionally unethically. Nonetheless, it would be appropriate not to title the study a randomized trial or use that term in the abstract. Instead, I recommend the authors use the same term employed in methods section of the study design: "Exploratory Study." The authors themselves call it that. Now they just need to make it clear up front.

***Response:*** *We agree that the fact that the trial was not registered is a weakness. It does not affect the weakness of this trial but we note that even now, large numbers of behavioural trials published in high quality journals are not pre-registered. We gave a great deal of consideration before submitting the first version as to whether to call this an RCT and in the end decided to do so because that is what it was. We have checked authoritative definitions (e.g. the UK’s National Institute for Health and Care Excellence) and they do not specify pre-registration in the definition. As the reviewer acknowledges, we make it very clear that the trial was not pre-registered. We believe that we must use the term RCT in the title etc but have added the word ‘exploratory’ to highlight this issue.*

1. Very low retention rate (7.5%) that appears to imbalanced by a relative rate of 24% between arms. This is a very unfortunate weakness that could have been avoided with some small and immediate incentives given that only one question is asked in the follow-up survey. Nonetheless, it is what it is. The level of bias from this extent of missing data (92.5%) cannot be overcome with imputation methods. And the argument that the relative difference (not absolute quit rate) is what matters for this study is undermined by the imbalanced retention rates. With 24% more data in one arm than the other, it very possible that the difference in quit rates is simply driven by the difference in retention rates.

***Response:*** *We agree that the low follow up is a major weakness and prevents estimation of absolute effect size. However, we disagree that the imbalance in follow-up rates could have contributed to the relative effect. As we explain in the introduction, we conducted an additional analysis only on those followed up and still got a highly significant effect with an odds ratio of 1.5. We have explored the issue of whether results from the follow-up only sample can possibly be affected by differential follow up rate and with both analytical reasoning and extensive modelling of extreme difference we find that it cannot. We were initially surprised but once we saw it, it was obvious.*

*To help see this, imagine a study of 1000 smokers with 500 allocated to each group. Then imagine 200 (40%) are followed-up in the intervention group and 100 (20%) in the control group. Imagine that in fact there is no difference in effect between the conditions so 50 (10%) are actually abstinent in each group. In an intent to treat analysis with missing equals smoking we would expect 20 (40% of 50) successes in the intervention group and 10 (20% of 50) in the control group which would translate to 20/500=4% in the intervention group versus 10/500=2% in the control group – apparently double the success rate in the intervention than control group which is clearly wrong. But in the follow-up only analysis the figures are 20/200=10% in the intervention group and 10/100=10% in the control group – correctly showing no effect. You can try this with any permutation of outcomes and it always comes out the same.*

 Moderate weaknesses

1. The main outcome of continuous abstinence after baseline is biased by differences in quit dates and simply does not give people enough time to even reach their quit date before they would already be counted a smoker. Thus, the actual quit rates could be higher. Its hard to say with this outcome. 

***Response:*** *We agree that the actual quit rates may well be higher. In fact, given what we observe in prospective studies of unaided quitting it would be surprising if the quit rates were not higher. We were not sure what the reviewer was referring to when saying that some participants may not have reached their quit dates by the follow up; the follow up was always 12 weeks after their designated quit date.*

1. Generalizablity is overstated for two reasons. The low retention rate makes the sample highly biased toward the most motivated people who are most likely to be reporting that they quit. Comparing the baseline characteristics of all those enrolled vs only those completed the outcome survey item would be very important and instructive about bias. The second reason it is overstated is that the sample is limited to those who have already chosen an app and this particular app to help them quit smoking. The sample age (29) is young for a mhealth smoking study, which typically is about age 40. 

***Response:*** *We now note in the discussion that the generalizability of the findings is limited by the low follow-up rate and to smokers. However, it should be noted that in the intention to treat analysis the sample was not limited to those followed up and the odds ratio was slightly higher than for the follow-up only sample and adjusting for baseline variables that are predictive of successful cessation also did not influence the finding. The data are available for anyone who would like to compare the baseline characteristics of those followed-up compared with who were not.*

# Peer review: Referee response for version 1

**DOI:** 10.5256/f1000research.17634.r38627

**Published:** 2018-10-08

**Authors:** Erica Cruvinel

**Affiliations:** 1 Preventive Medicine and Public Health Department, University of Kansas Medical Center, Kansas City, Kansas, USA

This manuscript is a study of the “Relative effectiveness of a full versus reduced version of the ‘Smoke Free’ mobile application for smoking cessation: a randomised controlled trial”. The use of such applications as described therein can extend the reach of smoking cessation interventions to a large number of smokers through low-cost approaches. Thus, the results of this study will be of great interest to the tobacco treatment community. I have several questions about the details of the study and some suggested edits. 

 Introduction:

 The introduction discusses most methodology decisions, such as those about the comparator group, follow up loss, analysis, and the measure of abstinence. But, it lacks some conceptual information. For instance, it does not address why the full version of the app was expected to be more effective than the reduced version. I would like to see a more conceptual description used as a background to support the development of an extended version beyond the app’s basic version. Also, I think methodology decisions would fit better under methods or study limitations. In addition, it is not clear what the authors mean by, “Also, in a trial where there is no greater psychological pressure to claim abstinence in one condition than another, use of self-report should not bias the estimated effect size“. I’m not sure if the authors refer to a non-superiority trial since the last sentence in the introduction session shows that they expected a better result in the intervention group. 

 Furthermore, I would like to have more information about the app itself. For instance, are people from all over the world able to download the app? In which languages is it available?

 Methods:

 Study design:

 Provide more details about the method used to generate the random allocation sequence (such as blocking and block size). 

 Participants:

 Were included daily and occasionally smokers? In which countries do the participants reside? Is the app only in English?

 Intervention and comparator 

 Show the theory that guided the development of these interventions in this section. 

 Measures:

 Did you collect data about how often the app was used and for how long?

 Analysis:

 How did you control against the duplication of data, such as if someone installed the app twice? 

 What strategy did you use to increase follow up response rates? Did you set up a reminder function to send notifications to the user to complete the follow-up questionnaire?

 How did you handle missing data? (details of any imputation method)

 Results:

 How did the participants use the app? If you included descriptive information from both groups, then it would help us to better understand participant interactions with the app.

 Did you have information about whether participants used other methods to quit smoking? For instance, nicotine replacement therapy?

 The sentence “Older participants, and in the MES analysis, those with longer time to their first cigarette of the day were more likely to report abstinence”. This interpretation is confusing because the results from participants who smoke cigarettes longer than 60 minutes were not significant. Also, those whose quit date was after the date of registration were less likely to report abstinence. What was the comparator?

 Table 2: It is not clear. What is the reference in table 2? 

 Discussion:

 The fifth sentence of the introduction states: “The odds ratios ranged between 1.50 and 1.90 in the two analyses and the percentage point differences ranged between 0.7% and 5.5%”. Is it not 0.85 and 0.7? 

 The last sentence in the third paragraph is not clear when looking at table 2. Which variable measured the difficulty of quitting?

 The quit rate in this study was lower compared to other studies (ex: BinDhim, McGeechan, Trevena, 2018 ^1^), as well as the follow-up rates. I would like to see more discussion about how the results from this study compare to those of other apps.

 The results showed that those using Android (versus iOS devices) were less likely to report abstinence. What’s the implication of this data?

I have read this submission. I believe that I have an appropriate level of expertise to confirm that it is of an acceptable scientific standard, however I have significant reservations, as outlined above.

## References

[1] BinDhimNFMcGeechanKTrevenaL: Smartphone Smoking Cessation Application (SSC App) trial: a multicountry double-blind automated randomised controlled trial of a smoking cessation decision-aid 'app'. *BMJ Open*.2017;8(1) : 2935841810.1136/bmjopen-2017-017105PMC5780681

# Response

**Published:** 2019-01-08

**Authors:** Robert West

**Affiliations:** University College London, UK

1. The introduction does not address why the full version of the app was expected to be more effective than the reduced version. I would like to see a more conceptual description used as a background to support the development of an extended version beyond the app’s basic version.

***Response:*** *We have now added this to the introduction and made reference to a paper that gives more detail.*

1. Methodology decisions would fit better under methods or study limitations.

**Response:** *We thought of doing this but in the end decided to keep it where it is because it made the methods section harder to follow and is part of the rationale for the study rather than what was actually done.*

1. It is not clear what the authors mean by, “Also, in a trial where there is no greater psychological pressure to claim abstinence in one condition than another, use of self-report should not bias the estimated effect size“. I’m not sure if the authors refer to a non-superiority trial since the last sentence in the introduction session shows that they expected a better result in the intervention group. 

***Response*** ***:*** *We obviously didn’t make this point clear. We were referring to the pressure that respondents might feel to falsely claim that they were abstinent. We have reworded this to try to make it clearer.*

1. I would like to have more information about the app itself. For instance, are people from all over the world able to download the app? In which languages is it available?

***Response:*** *We have now added this information. It is available globally but only in the English language.*

1. Provide more details about the method used to generate the random allocation sequence (such as blocking and block size). 

***Response:*** *We now included this information. The app generated a random number (1 or 2) with equal probability during the registration process for each user.*

1. Were included daily and occasionally smokers?

***Response:*** *As indicated in the paper all smokers were included. We now make it explicit that this included non-daily smokers.*

1. Show the theory that guided the development of these interventions in this section. 

***Response:*** *We now include this in the explanation as to why we thought the full intervention would be more effective than the reduced version.*

1. Did you collect data about how often the app was used and for how long?

***Response:*** *We did not collect information about number of times the app was used, unfortunately.*

1. How did you control against the duplication of data, such as if someone installed the app twice?

***Response:*** *We already explain that if the app was downloaded more than once on the same device, we used the first occurrence only. Please see page no 5, paragraph 4 (continued on page 6).*

  

1. What strategy did you use to increase follow up response rates? Did you set up a reminder function to send notifications to the user to complete the follow-up questionnaire?

***Response:*** *We already explain that push notifications appeared on the home screen of the device to solicit responses to the follow up questionnaire. We now make clear that reminders were not sent. Please see page no 7, paragraph 4.*

1. How did you handle missing data (details of any imputation method)?

***Response:*** *We attempted to make this clear in the paper. We used both the traditional ‘missing equals smoking’ method and follow-up only. The aim was to address different types of bias that may arise from these two methods. As noted by reviewer 1, more sophisticated imputation methods would have required much greater follow-up rates.*

1. How did the participants use the app? If you included descriptive information from both groups, then it would help us to better understand participant interactions with the app.

***Response:*** *Unfortunately* *we do not have this information.*

1. Did you have information about whether participants used other methods to quit smoking? For instance, nicotine replacement therapy?

***Response:*** *Unfortunately not. It possible that participants in the intervention group used other methods to help them quit and indeed part of the effect of the app may have been to get them to do so since advice on medication use was one of the behaviour change techniques used.*

1. The sentence “Older participants, and in the MES analysis, those with longer time to their first cigarette of the day were more likely to report abstinence”. This interpretation is confusing because the results from participants who smoke cigarettes longer than 60 minutes were not significant.

***Response:*** *We have rephrased this to make clear that the association was only statistically significantly for the middle two categories of time to first cigarette.*

1. Also, those whose quit date was after the date of registration were less likely to report abstinence. What was the comparator?

***Response:*** *We now make clearer that the comparator was those whose quit date was the same as the date of registration.*

1. Table 2: It is not clear. What is the reference in table 2?

***Response:*** *This is the standard way to report results from logistic regression analyses with categorical independent variables. The ‘reference’ is the group against which the other groups are compared.*

1. The fifth sentence of the introduction states: “The odds ratios ranged between 1.50 and 1.90 in the two analyses and the percentage point differences ranged between 0.7% and 5.5%”. Is it not 0.85 and 0.7?

***Response:*** *We think the reviewer means the second sentence in the discussion but were still not sure what to make of this comment. This sentence just summarises the results from the comparison between the intervention and comparator conditions as stated in the results. However, to make this clearer we have rephrased this sentence.*

1. The last sentence in the third paragraph is not clear when looking at table 2. Which variable measured the difficulty of quitting?

***Response:*** *We now make clearer that this was based on using baseline variables that predicted abstinence.*

1. The quit rate in this study was lower compared to other studies (ex: BinDhim, McGeechan, Trevena, 2018), as well as the follow-up rates. I would like to see more discussion about how the results from this study compare to those of other apps.

***Response:*** *As noted by Reviewer 1, the follow-up rate was too low to be able to gain an accurate estimate of absolute quit rates. However, we now included discussion of how the results compare with those of previous RCTs of smoking cessation apps.*

1. The results showed that those using Android (versus iOS devices) were less likely to report abstinence. What’s the implication of this data?

***Response:*** *We now include a brief discussion of this finding.*
